# Electrochemical Immunosensor for Ultra-Low Detection of Human Papillomavirus Biomarker for Cervical Cancer

**DOI:** 10.1021/acssensors.3c00677

**Published:** 2023-06-29

**Authors:** Siwaphiwe Peteni, Okoroike C. Ozoemena, Tobile Khawula, Aderemi B. Haruna, Frankie J. Rawson, Leshweni J. Shai, Oluwafunmilola Ola, Kenneth I. Ozoemena

**Affiliations:** † Molecular Science Institute, School of Chemistry, University of the Witwatersrand, Johannesburg 2050, South Africa; ‡ School of Pharmacy, Biodiscovery Institute University of Nottingham, Nottingham NG7 2RD, U.K.; § Department of Biomedical Sciences, Tshwane University of Technology, Pretoria 0001, South Africa; ∥ Advanced Materials Group, Faculty of Engineering, The University of Nottingham, Nottingham NG7 2RD, U.K.

**Keywords:** onion-like carbon, polyacrylonitrile fiber, antigenic HPV-16 L1 peptide, anti-HPV-16 L1 antibody, ovalbumin protein, anti-ovalbumin antibody, ultra-low detection

## Abstract

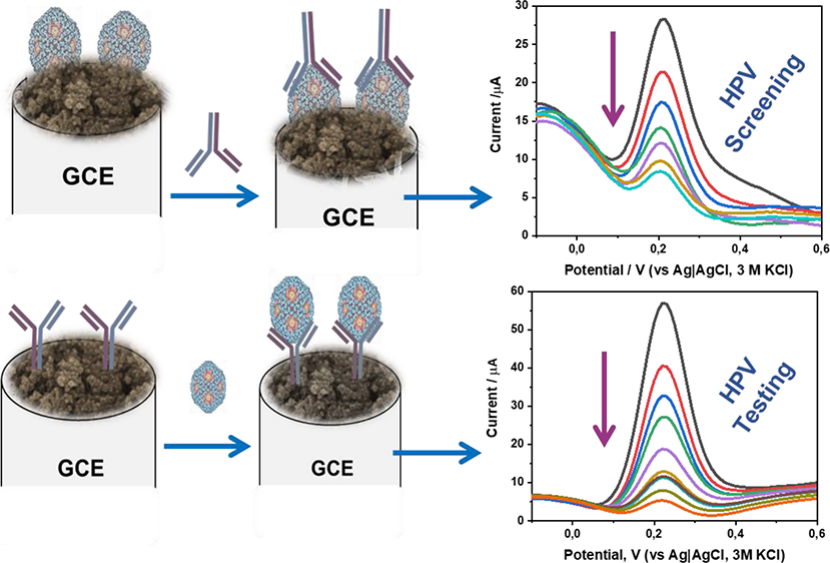

Human papillomavirus (HPV) is the causative agent for cervical cancer. Of the various types of HPV, the high-risk HPV-16 type is the most important antigenic high-risk HPV. In this work, the antigenic HPV-16 L1 peptide was immobilized on a glassy carbon electrode and used to detect several concentrations of the anti-HPV-16 L1 antibody, and vice versa. Two electrode platforms were used: onion-like carbon (OLC) and its polyacrylonitrile (OLC-PAN) composites. Both platforms gave a wide linear concentration range (1.95 fg/mL to 6.25 ng/mL), excellent sensitivity (>5.2 μA/log ([HPV-16 L1, fg/mL]), and extra-ordinarily low limit of detection (LoD) of 1.83 fg/mL (32.7 aM) and 0.61 fg/mL (10.9 aM) for OLC-PAN and OLC-based immunosensors, respectively. OLC-PAN modified with the HPV-16 L1 protein showed low LoD for the HPV-16 L1 antibody (2.54 fg/mL, i.e., 45.36 aM), proving its potential use for screening purposes. The specificity of detection was proven with the anti-ovalbumin antibody (anti-OVA) and native ovalbumin protein (OVA). An immobilized antigenic HPV-16 L1 peptide showed insignificant interaction with anti-OVA in contrast with the excellent interaction with anti-HPV-16 L1 antibody, thus proving high specificity. The application of the immunosensor as a potential point-of-care (PoC) diagnostic device was investigated with screen-printed carbon electrodes, which detected ultra-low (ca. 0.7 fg/mL ≈ 12.5 aM) and high (ca. 12 μg/mL ≈ 0.21 μM) concentrations. This study represents the lowest LoD reported for HPV-16 L1. It opens the door for further investigation with other electrode platforms and realization of PoC diagnostic devices for screening and testing of HPV biomarkers for cervical cancer.

According to WHO statistics for 2018, cervical cancer alone has killed about 375,000 women and girls out of 500,000 cases, most of these cases are from Asia, Latin America, and sub-Saharan Africa.^1^ Although vaccination is one of the preventative ways in which one may follow, there is a problem with the rate of uptake, and it is also advisable to be vaccinated at a younger age for more effectiveness. Human papillomaviruses (HPV) are the causative agents of cervical, anal, and vaginal cancers to name a few. The human papillomavirus L1 protein (HPV-L1) is found in 90% of the HPV capsid and is known to be directly involved in the process of the HPV infection of host cells.^2^ These viruses are grouped as low-risk or high-risk depending on the type of lesion they cause. The low-risk HPVs are known to be benign and, in most cases, can be cleared by the body without any grievous harm, while the high-risk HPVs are associated with malignancy, which is responsible for the cancers mentioned above.^3^ Of the 14 known HPV types, the high-risk HPV-16 and -18 are mostly associated with cancer of the cervix. HPV-16 and -18 are responsible for the encoding of two oncogenes, E6 and E7, which are responsible for the progression to cancer.^4−6^ Both high-risk HPV-16 and HPV-18 are thought to be responsible for 93–100% of cervical cancer cases,^7^ with HPV-16 being the most dominant viral strain that affects the cervix, followed by the HPV-18 strain.

The persistence of HPV infection leads to the so-called cervical intraepithelial neoplasia (CIN). If detected early (i.e., when the dysplatic cells are confined within the surface epithelium of the cervix), the CIN can easily be cured. If not detected at the early stage, they can penetrate the basement of the membrane to become invasive cervical cancer and spread into the nearby organs, e.g., the uterus, bladder, rectum, and the pelvic lymph nodes, thereby causing death. The mortality rate of cervical cancer in the poorest countries has been estimated as twofold higher than for the wealthy countries.^1^ In many low- and middle-income earning countries (LMICs), no organized cervical cancer screening programs exist. LMICs bear the largest burden of human immunodeficiency virus (HIV) infection while persistent high-risk HPV infection is more common among HIV-infected women. Thus, the risk of cervical cancer is increased in women with HIV/AIDS. This underscores the urgent need for the development of low-cost diagnostic devices for HPV.

There are several diagnostic methods for the detection of HPV,^8^ which include Papanicolaou (Pap) smear (aka cervical or vaginal cytology), direct visual inspection of the cervix with acetic acid (VIA) or iodine (VILLI), polymerase chain reaction (PCR), enzyme-linked immunosorbent assay (ELISA), radioimmunoassay (RIA), immunohistochemistry (IHC), and flow cytometry (FC). These methods suffer from several shortcomings. For example, the Pap smear which is the most frequently used diagnostic method has reduced the mortality rate of cervical cancer by up to 50%; however, it is fraught with poor sensitivity (between 50 and 60%).^9^ Pap smear is not sustainable in under-resourced LMIC settings with low socio-economic and educational level, limited skilled cyto-screener and cytopathology workforce^10^ and where, despite a high prevalence of cervical cancer, lack of follow-up and poor adherence to treatment are major impediments for program success. The low-cost VIA and VILLI methods require a trained workforce to deliver and are characterized by poor specificity, which can result in the substantial over-treatment of patients. In addition, it is very difficult to detect small ectocervical and endocervical lesions under visual inspection so, with a once-in-a-lifetime screen or wide screening intervals, pre-cancer can be missed and progress to invasive cancer. PCR, ELISA, RIA, IHC, and FC are efficient but have the disadvantages that they are invasive, expensive, time-consuming, and bulky (i.e., limited to large hospitals).

Considering that the Pap smear method is the most employed technique for the detection of HPV infection in the LMICs, there is a need to design and develop a diagnostic method that complements it, but with advantages of better sensitivity and selectivity (since the level of antibodies in both symptomatic and asymptomatic patients is generally very low), less invasive, and easier to use (preferably self-use) to allow for increased coverage in the communities of the LMICs. Electrochemical detection methods meet the above criteria: they are characterized by their simplicity, high sensitivity, and selectivity and, importantly, electrochemical sensors can be miniaturized (smaller devices) for hand-held, point-of-care (PoC) diagnostic testing and screening devices that require little training for operation in a given community setting.

Today, most of the electrochemical methods of detecting HPV have been focused on nucleic acid (DNA) sensors.^11−14^ To our knowledge, the use of protein biomarkers for the electrochemical detection of HPV is largely unknown. There are only a few reports that attempted the utilization of HPV protein biomarkers.^13,15−18^ For example, Piro et al.^15^ reported the use of a very complex conjugated electro-co-polymer to encapsulate the antigenic HPV-16 L1 on a glassy carbon electrode (GCE) and used it to detect just a single concentration of the anti-HPV-16 L1 antibody. Second, Valencia et al.^16^ reported the detection of anti-peptide antibodies of HPV-L1 by using a gold electrode modified with a peptide (SPINNTKPHEAR) derived from the HPV-L1, a technique that involved a lot of preparation steps and only tested or suited for the detection of anti-HPV in serum samples (screening). Recently, Wang et al.^17^ used quasi-spherical Ag@Au core–shell nanoparticles on graphene oxide (Ag@AuNPs-GO) as the current response amplifier for the detection of HPV-16 L1. To close the knowledge gap, this work used recombinant HPV-16 L1 protein (HPV-16) and anti-HPV16 L1 antibody (anti-HPV) for the fabrication of an electrochemical immunosensor with the potential application in the screening (i.e., for asymptomatic people) and diagnostic tests (i.e., for symptomatic people) of HPV-16. It is known that the HPV-L1 protein is the dominant epitope (present in 90% of the HPV capsid) directly involved in the process of HPV infection in patients as well as in the prophylactic vaccines, thus the use of the high-risk HPV-16 and anti-HPV-16 is of high importance. We show that polyacrylonitrile (PAN) fiber modified with onion-like carbons (OLC-PAN) nanocomposite represents a viable electrode platform for the encapsulation of the anti-HPV-16 antibodies for the detection of HPV-16 antigen (i.e., testing), and for the encapsulation of the HPV-16 antigen for the detection of the anti-HPV-16 antibody (i.e., screening). OLC is a relatively new carbonaceous material that has been applied as an excellent electrode modifier for electrocatalysis,^19,20^ electrochemical sensing,^21−23^ and electrochemical energy storage^24,25^ due to its attractive properties of high specific surface area, high conductivity, and electrocatalytic properties. In addition, PAN is easy to synthesize and demonstrates good absorptivity for pollutants.^26^ Although it is a poor conductor, it has shown better electrochemistry when modified with OLC,^23^ such that its hybrid has better properties than the individual materials. In addition, from a previous study,^23^ it was found that although OLC has high conductivity and high specific surface area, it showed poor electrochemical responses hence a hybrid material was synthesized and tested.

## Results and Discussion

### SEM and TEM Characterization

**Figure 1:**
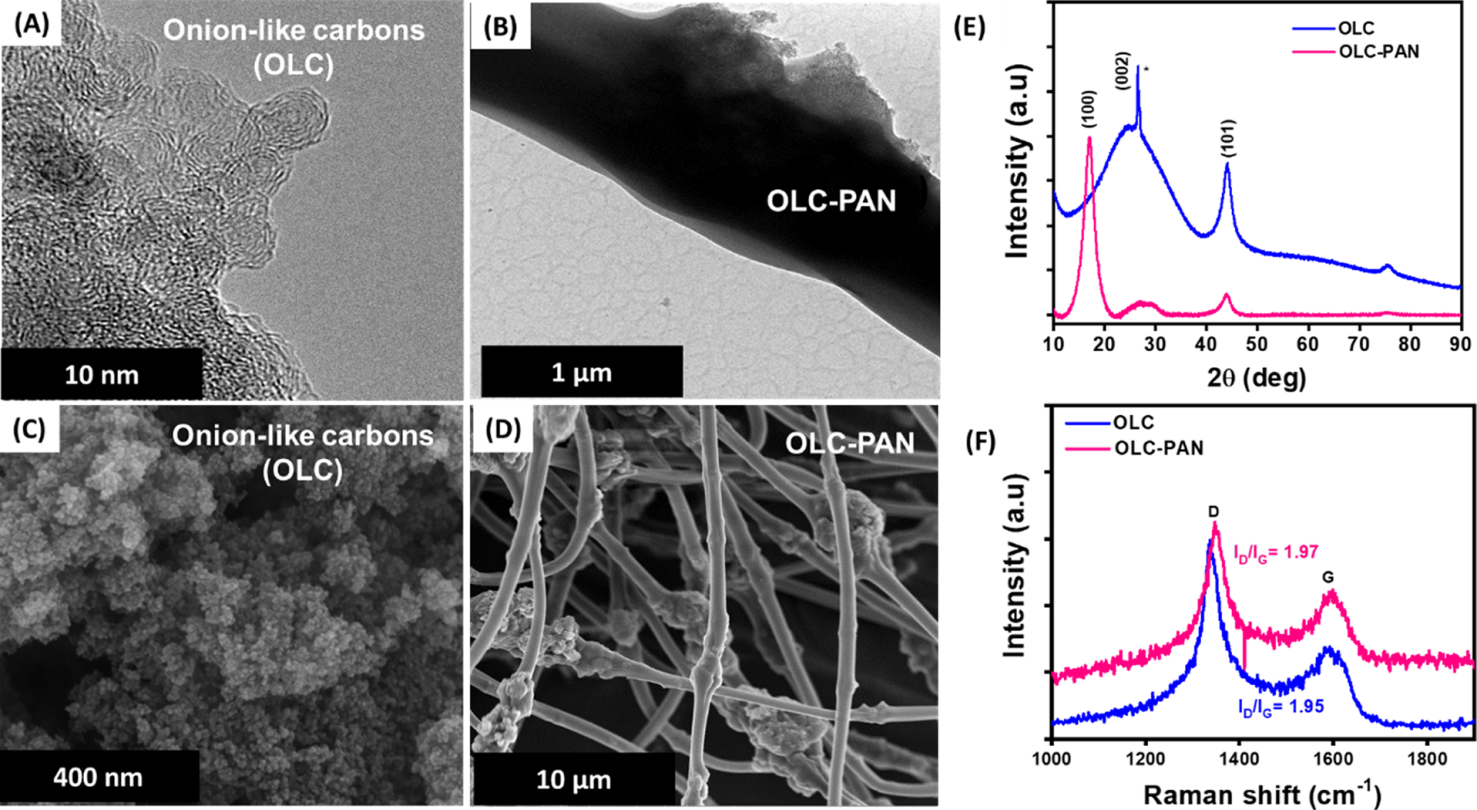
TEM images of (A) OLC and (B) OLC-PSAN, SEM images of (C) OLC and (D) OLC-PAN, (E) XRD patterns of powdered OL and OLC-PAN, and (F) Raman spectra of OLC and OLC-PAN.

The physicochemical characterizations such as TEM, SEM, XRD, Raman, and surface analysis are discussed in this section. HRTEM images are shown in Figure 1A for OLC and Figure 1B for OLC-PAN. The HRTEM image of OLC is interconnected graphitic layers, which resembles onion-like rings with the outside layers connected to form an oval-like shape, while the OLC-PAN does not show any distinct shape. The SEM images of **OLC** and **OLC-PAN** are shown in Figure 1C,D, respectively. The morphology of OLC is agglomerates of nanoparticles, while the OLC-PAN shows rough and smoothened surfaces, the roughness of the PAN surface confirms the successful incorporation of the OLC into the PAN matrix.

### X-ray Diffraction, Raman Spectroscopy, and Specific Surface Area Analysis

Figure 1E compares the powder XRD patterns of the OLC and OLC-PAN. The OLC shows two distinct peaks at 2θ of ∼26.4 and 44.0° corresponding to the amorphous (i.e., (002) plane) and crystalline graphite (i.e., (101) plane), respectively. The OLC-PAN, on the other hand, shows the same peaks as the OLC, but with an extra peak at 2θ = 17.1° that corresponds to the (100) plane of the polymer. The XRD of the OLC-PAN confirms the presence of the OLC. Figure 1F compares the Raman spectra of the OLC and OLC-PAN. Both **OLC** and **OLC-PAN** show the two characteristic distinct D (defect) bands due to the out-of-plane vibrations of sp3-bonded carbons, and G (graphitic) band arising from the in-plane vibrations of the sp3-bonded carbons. The D and G bands were observed at 337.4 and 1593 cm^–1^ for the OLC, respectively, while those of the OLC-PAN appear at 1347.9 and 1598.2 cm^–1^, respectively. The degree of graphitization was determined using the conventional *I*_D_/*I*_G_ ratio. The *I*_D_/*I*_G_ ratio of **OLC-PAN** was 1.97 compared to the OLC of 1.95, indicating that the OLC-PAN is slightly more defective than the OLC alone.

The BET surface area, pore volume, and pore size were calculated as 279.05 m^2^g^–1^, 1.20 cm^3^g^–1^, and 17.11 nm, respectively for OLC, and as 117.69 m^2^g^–1^, 0.54 cm^3^g^–1^, and 22.89 nm, respectively, for OLC-PAN. The decrease in the surface area of **OLC-PAN** compared to the OLC should be expected considering the bulky nature of the OLC-PAN as evident from the SEM and TEM images.

### X-ray Photoelectron Spectroscopy

**Figure 2:**
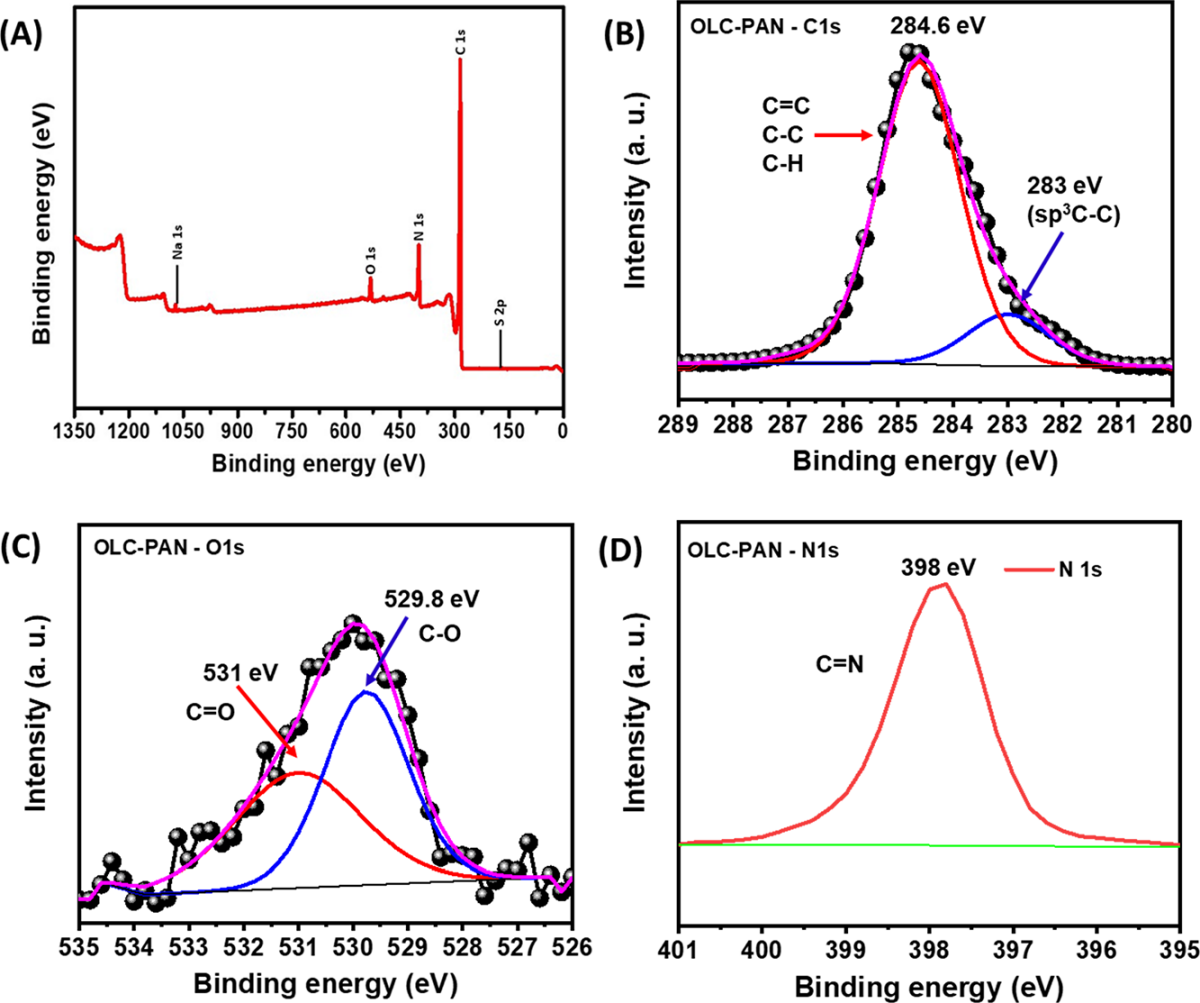
(A) Wide scan spectrum of OLC-PAN, and deconvoluted spectra of (B) C1s, (C) O1s, and (D) N1s of OLC-PAN.

Figure 2 shows the XPS wide scan spectrum of the OLC-PAN (Figure 2A) with the expected peaks for C1s, O1s, and N1s at binding energies of 286, 397, and 536 eV, respectively. The deconvoluted spectra are shown for C 1s (Figure 2B), O 1s (Figure 2C), and N 1s (Figure 2D). The C1s peak was deconvoluted to two binding energies at 283 eV (sp^3^(C–C), defects) and 284.6 eV (C–C). It is to be noted that the sp^3^(C–C) and sp^2^(C–C) defect peaks are characteristics of the nanodiamonds,^27,28^ and the correct peak assignment has been a subject of controversy; most researchers believe that the peak position of sp^2^C is lower than that of the sp^3^C, few workers believe the opposite.^29^ Interestingly, however, it has recently been demonstrated (via calculations) that the peak position of sp^2^C is greater than that of the sp^3^C, which agrees with the experimental results of those few workers.^29^ According to these workers,^29^ the reasons for the ambiguous assignments could be related to charging effects and defects (such as pentagons, heptagons, and functional groups) present in diamonds. The values of the sp^3^(C–C) range between 283.15 and 283.21 eV,^29^ and even at 282.8 eV^30^ which are in close agreement with our value of 283 eV. Importantly, considering that the OLC used in this study was obtained from detonation nanodiamonds (DNDs), we believe that the observed peak at 283 eV (sp^3^(C–C)) could have arisen from the unconverted DNDs present in the OLC. Similarly, the O1s peak was deconvoluted with two binding energies at 531.2 eV (C=O) and 529.8 eV (C–O) (Figure 2C). These peak assignments agree with previous reports.^31,32^ In addition, the N 1s was deconvoluted to one peak at 398 eV corresponding to a cyanide (C=N) bond arising from the PAN component of the composite structure. Note that the two binding energies of the O 1s peak as well as that of the N 1s are slightly shifted to lower values, but this can be attributed to electron transfer from the PAN to the oxygenated functional groups of the OLC.

### Electroanalytical Performance of the Immunosensors Toward HPV-16 L1 Detection

**Figure 3:**
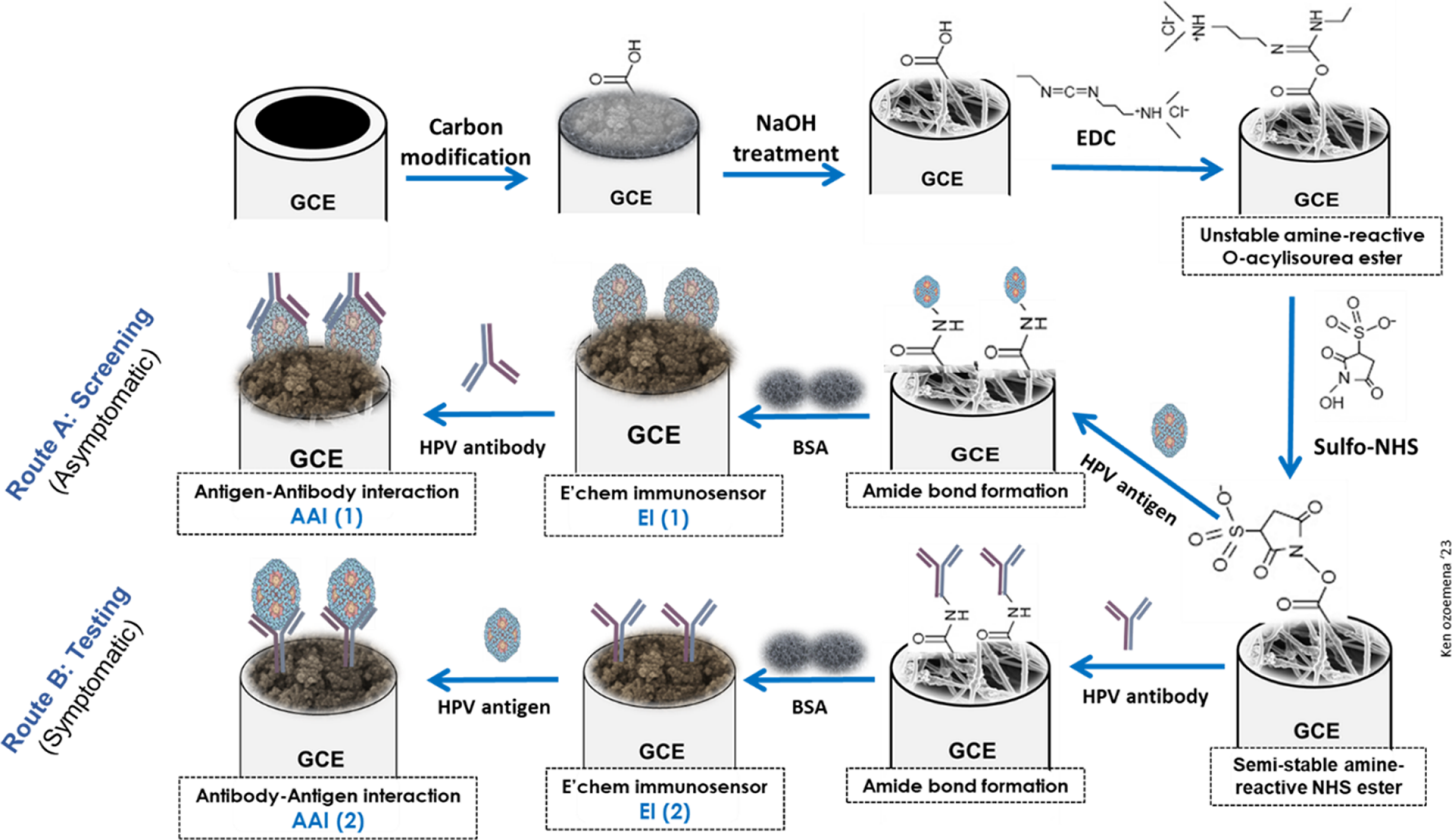
Typical fabrication pathway for HPV-16 L1 immunosensors. The reagents in each path are identified in the Experimental Section.

The fabrication of the HPV-16 L1 immunosensors and antigen–antibody interaction mechanisms are described in the Experimental Section but summarized as shown in Figure 3. The HPV antigen or antibody is immobilized onto the OLC- or OLC-PAN electrode via the conventional covalent bonding process through the formation of the strong amide bond using the sulfo-NHS ester.^23,33−35^ In practice, route A describes the screening of HPV-16 infection (asymptomatic individuals), while route B is for the testing of the HPV-16 infection (in individual who presents some symptoms).

Cyclic voltammetric evolutions of the bare GCE, the electrochemical immunosensors (EI (1) and EI (2), Figure 3), and their corresponding antigen–antibody interaction stages (AA(I) and AAI (2), Figure 3) were conducted in the redox probe solution (i.e., PBS/AE, pH 7.4, containing 0.1 mM [Fe(CN)_6_]^4–^/[Fe(CN)_6_]^3–^) (see Figure S1). It was observed that the OLC-PAN-immunosensor exhibited the strongest current suppression (capacitive behavior) upon interaction with the analytical HPV-16 antigen than the OLC-based counterpart. The difference between the two immunosensor electrodes may be related to the conductivity of the electrode platforms, where OLC shows higher electronic conductivity than the OLC-PAN counterpart.

**Figure 4:**
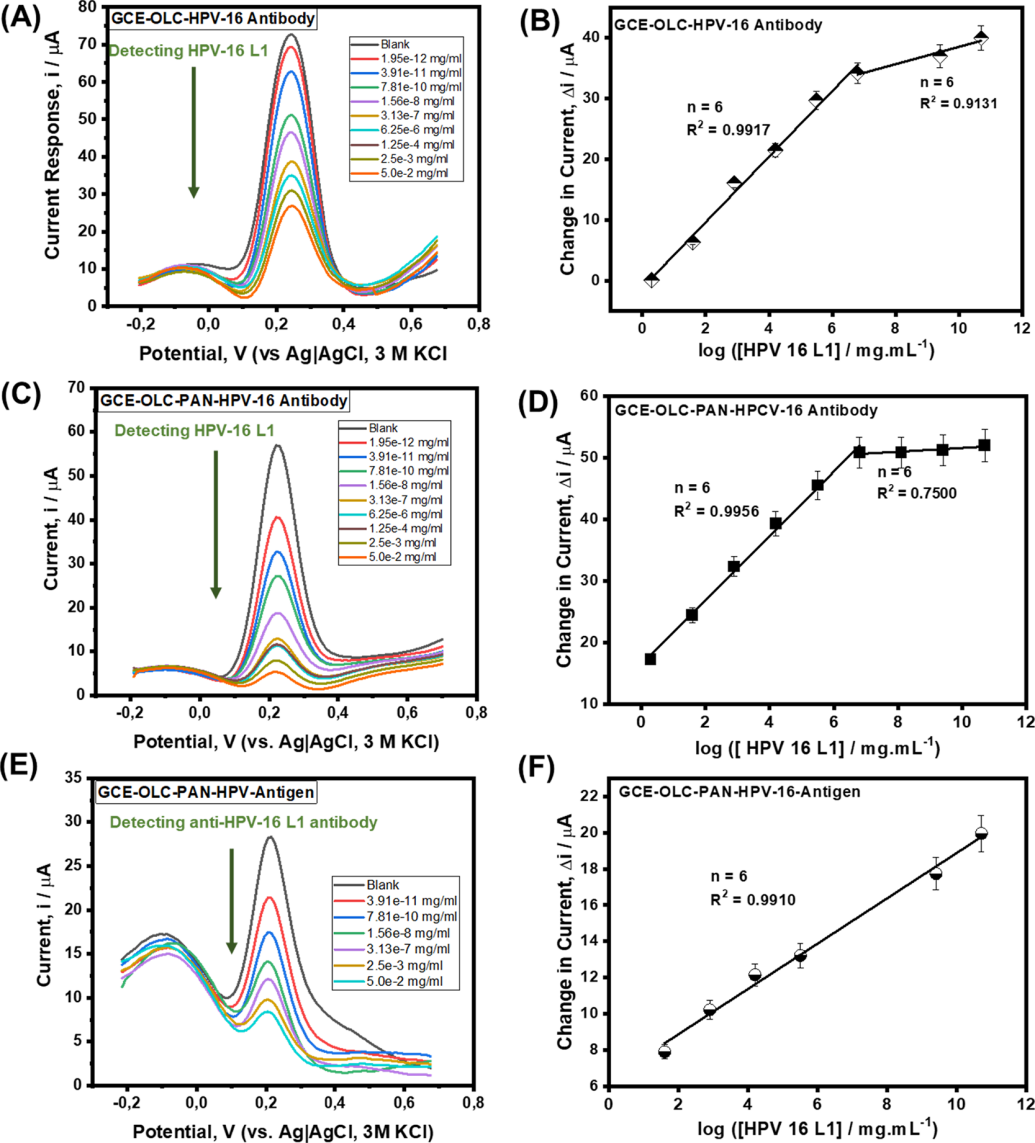
Square wave voltammetric detection. (A) Concentration studies of antigenic HPV-16 L1 protein (Ag) on OLC-based immunosensor and (B) corresponding linear plot of the changes in current vs log [ HPV-16 L1]; (C) concentration studies of antigenic HPV-16 L1 protein (Ag) on OLC-PAN-based platform and (D) corresponding linear plot of the changes in current vs log [ HPV-16 L1]; (E) concentration studies of anti-HPV 16 L1 antibodies on OLC-PAN-based immunosensor and (F) corresponding linear plot of the changes in current vs log [anti-HPV 16 L1]. Please see the Experimental Section for details.

The developed electrochemical immunosensors were employed to detect HPV-16 L1 antigen at different concentrations (Figure 4). The concentration studies were conducted by exposing/immersing the immunosensor in different concentrations of the HPV-16 L1 antigen (from 1.95 × 10^–12^ to 5.0 × 10^–2^ mg/mL) for 5 min to allow for the complexation to occur, after which square wave voltammetry (SWV) was used to monitor the reaction in the presence of the redox probe. The continuous suppression of the redox peaks of the redox probe upon increasing concentration of the HPV-16 L1 antigen is indicative of the excellent complexation between Ab and Ag. The OLC-based immunosensor (Figure 4A) showed a broader peak response compared to its OLC-PAN counterpart (Figure 4C). Aside from this disadvantage of the OLC-based electrode platform, the OLC-PAN-based immunosensor also showed better linearity than the OLC-based immunosensor (i.e., *R*^2^ = 0.9971 cf 0.9823). Thus, the OLC-PAN electrode was used to immobilize the HPV-16 L1 antibody for the detection of the HPV-16 L1 antigen. The current response for each concentration was determined by taking the difference from the blank, i.e., (Δ*i*/μA). For both immunosensors, two linear concentration ranges (Figure 4B,C, for OLC and OLC-PAN-based immunosensors, respectively) were observed, at low (i.e., 1.95 × 10^–12^–6.25 × 10^–6^ mg/mL) and high (6.25 × 10^–6^–5.0 × 10^–2^ mg/mL) concentration ranges.

Considering that the OLC-PAN-based electrode platform gives a sharper current response The results are summarized as follows:

#### OLC-Based Immunosensors for HPV-16 Antigen Detection


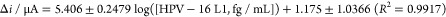
1

#### OLC-PAN-Based Immunosensors for HPV-16 Antigen Detection


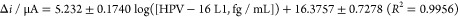
2

#### OLC-PAN-Based Immunosensors for HPV-16 Antibody Detection


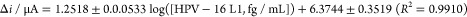
3

As summarized in Table 1, the immunosensors exhibited wide linear concentration ranges (1.95 fg/mL to 6.0 ng/mL, i.e., 34.82 aM to 0.11 nM), excellent sensitivity at the low concentration range for the detection of antigen (i.e., 5.406 ± 0.2479 μA/log([HPV-16 L1, fg/mL]) for the OLC-based immunosensor, and 5.232 ± 0.1740 μA/log([HPV-16 L1, fg/mL]) for the OLC-PAN-based immunosensor). For the detection of the antibody at OLC-PAN-based immunosensor, the sensitivity is lower than observed for the antigen (i.e., 1.2518 ± 0.0533 μA/log([HPV-16 L1, fg/mL]). The immunosensors showed ultra-low detection limits (i.e., LoD = 3 *s/m*, and LoQ = 10 *s/m*, where *s* is the standard deviation of the intercept, while *m* is the slope/sensitivity of the calibration plot). The values were calculated as the average of six electrodes and are summarized in Table 1, with LoD as ca. 0.61 fg/mL (10.89 aM) and 1.83 fg/mL (32.7 aM) for OLC-PAN and OLC-based immunosensors, respectively. Using the OLC-PAN-based immunosensor, the HPV-16 L1 antibody was detected at 2.54 fg/mL (45.36 aM). The extraordinary low detection limits shown in this work represent the first of its kind for the detection of HPV-16 L1 using either immunosensor or nucleic acid-based sensors (Table 1).

**Table 1:** Comparison of Various Electrochemical and Non-electrochemical Detection Methods for HPV16 L1^a^

platform/modifier	target	technique	LCR	LoD	LoQ	ref
GCE-OLC	HPV-16 L1 (antigen)	SWV	1.95 fg/mL–6.25 ng/mL (34.2 aM–0.112 nM)	1.83 fg/mL (32.7 aM)	6.11 fg/mL (0.11 fM)	this work
GCE-OLC-PAN	HPV-16 L1 (antigen)	SWV	1.95 fg/mL–6.25 ng/mL (34.2 aM–0.112 nM)	0.61 fg/mL (10.89 m aM)	2.03 fg/mL (36.3 aM)	this work
GCE-OLC-PAN	HPV-16 L1 (antibody)	SWV	39.1 fg/mL–60 μg/mL (0.70 fM–1.07 μM)	2.54 fg/mL (45.36 aM)	8.46 fg/mL (0.15 fM)	this work
prGO-MoS_2_	HPV-16 L1 (antigen)	DPV	3.5–35.3 pM	1.75 pM		(13)
GC-polymer	HPV-16 L1 (antigen)	SWV		50 nM		(15)
Ag@AuNPs-GO/SPA	HPV-16 L1 (antigen)	DPV	0.005–400 ng/mL	2.0 pg./mL		(17)
Pt/PaN/MWCNTs	HPV-16 L1 (antigen)	CV/SWV	10–80 nM	490 pM		(18)
melamine-AuNPs	HPV-16 L1 (antigen)	LDI MS	2–80 ng /mL	58.8 pg/mL		(40)
AuNPs-Aptamer	HPV-16 L1 (antigen)	UV–vis/colorimetry	9.6–201.6 ng /mL	9.6 ng/mL		(41)

a KEY: Molar mass of HPV-16 L1 (56 kDa) was used to convert the gravimetric concentrations to their corresponding molar concentrations. LCR (linear concentration range); LoD (limit of detection); LoQ (limit of quantification); PaN (polyaniline); AuNPs (gold nanoparticles); SPA (staphylococcal protein A); LDI MS (laser desorption ionization mass spectrometry); SWV (square wave voltammetry); and DPV (differential pulse voltammetry).

For possible clinical application, we can compare with the conventional liquid-based pap test (LBPT) (which is still the most easily accessible technique in rural areas of resource-limited countries, or LMICs) and the more advanced PCR-based techniques. In LBPT, the specimen sample is rinsed into a sample container, the liquid is placed onto a microscope slide and then examined under a microscope to check if the cells are normal or not. The detection limit of LBPT is about 2000 copies/mL^36^ (i.e., 4.04 fg/mL). There is a commercial APTIMA HPV assay that detects the mRNA of high-risk HPV including HPV 16 L1 was reported to be able to detect 106 mRNA copies/reaction (i.e., ca. 0.21 fg). The real-time PCR (RT-PCR) is the most important molecular technique for testing high-risk HPV types in clinical laboratories, especially in high-income settings and advanced countries. In addition, recombinase polymerase amplification (RPA) represents an isothermal alternative to the PCR. Previously, Hesselink et al.^37^ showed that the HPV-risk assay (i.e., a type of real-time PCR assay) used for testing clinical samples gave LoD of 460 copies/reaction (ca. 0.93 fg) for the HPV-16 L1 genotype). In addition, in 2023, Wongsamart et al.^38^ reported an advanced form of RPA known as the multiplex recombinase polymerase amplification (mRPA), which gave an LoD of 1000 copies/reaction (i.e., 2.01 fg) for the HPV-16 L1 genotype. The LoD values of our proposed electrochemical immunosensor are lower than the conventional LBPT, and comparable or even lower than the advanced PCR and mRPA techniques. It should be noted, however, that we could not subject our immunosensors to testing real clinical samples at this time due to the inherent difficulty of obtaining ethical clearance for human samples, but efforts are being made to overcome this in the future.

### Specificity Studies and Sensor Reusability

**Figure 5:**
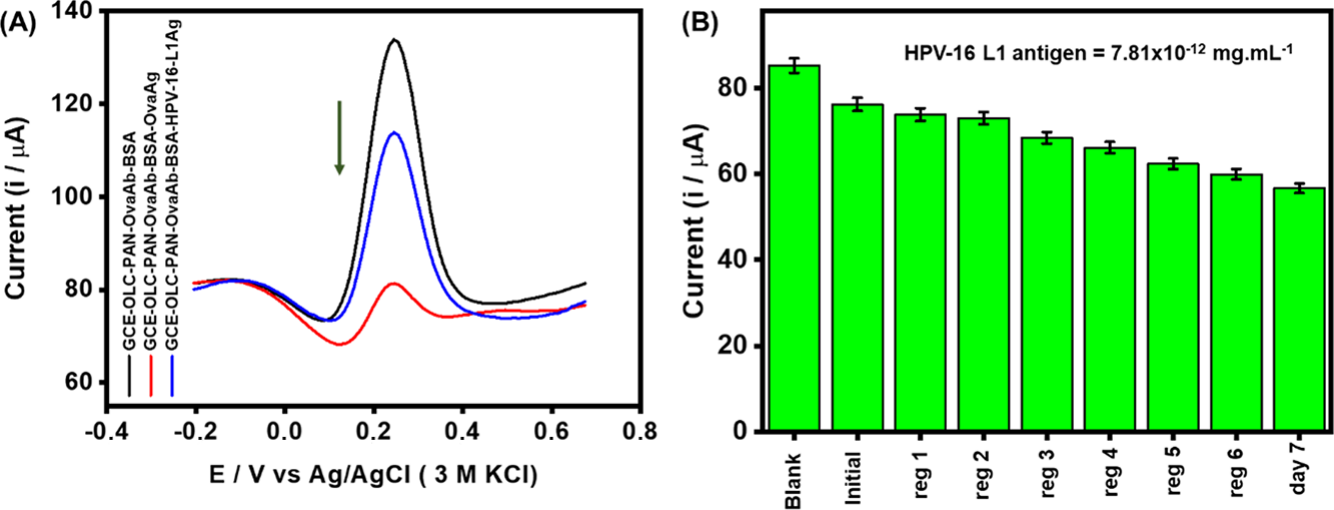
(A) Square wave voltammetric responses of GCE-OLC-PAN-OVAAb-BSA toward OVA-antigen (7.8 × 10^–15^ fg/mL) and HPV-16 L1 antigen (7.8 × 10^–15^ fg/mL), (B) Bar chart describing the responses of HPV-16 L1 antibody toward HPV-16 L1 (7.8 × 10^–15^ fg/mL) before (initial) and after six regeneration steps (reg 1 – reg 6) on day 1 and day 7. At every regeneration step, the detection of HPV-16 L1 antigen was repeated five times.

To test for specificity, we adopted the use of an anti-ovalbumin antibody (anti-OVA) and native ovalbumin protein (OVA) as reported elsewhere.^15^ The anti-OVA (Ab) was immobilized as prescribed during the experimental session and the anti-OVA immunosensor. The constructed immunosensor (GCE-OLC-PAN-OVAAb-BSA) was used to compare the extent to which the OVA-antibody can interact with the corresponding OVA-antigen and HPV-16 L1 antigen of similar concentration (7.8 × 10^–15^ fg/mL). As shown in Figure 5A, the OVA-antibody exhibited very strong interaction with the OVA-antigen but with weak or no interaction with the HPV-16 L1 antigen. The rejection of the OVA-ab by the HPV-16 L1 antigen confirms the specificity of the HPV-16 L1 antigen with its corresponding antibody.

To test for reusability/reproducibility of the proposed immunosensor, we adopted the well-established procedure of glycine chemistry.^39^ In a nutshell, the ability to reuse OLC-PAN-based immunosensor after chemically stripping off its active HPV-16 L1 antibody was interrogated using a constant concentration of the HPV-16 L1 antigen (7.8 × 10^–15^ fg/mL). On the first day, the immunosensor was used to detect the antigen, thereafter the bound antigen was stripped off by dipping the immunosensor into a buffer solution of glycine HCl (pH 2.8) for 5 min and then reused to detect the HPV-16 L1 antigen. The detection–stripping cycle was carried out six different times with each step used to detect the antigen five times. After the repetitive regeneration-detection steps, the immunosensor electrode was stored in the refrigerator at 4 °C, and the process was repeated on the 7th day.

As shown in Figure 5B, the immunosensor was found to be reusable even on the 7th day, albeit with minor loss in the current response compared to the 6th regeneration-detection step on the first day.

### Proof of Concept

**Figure 6:**
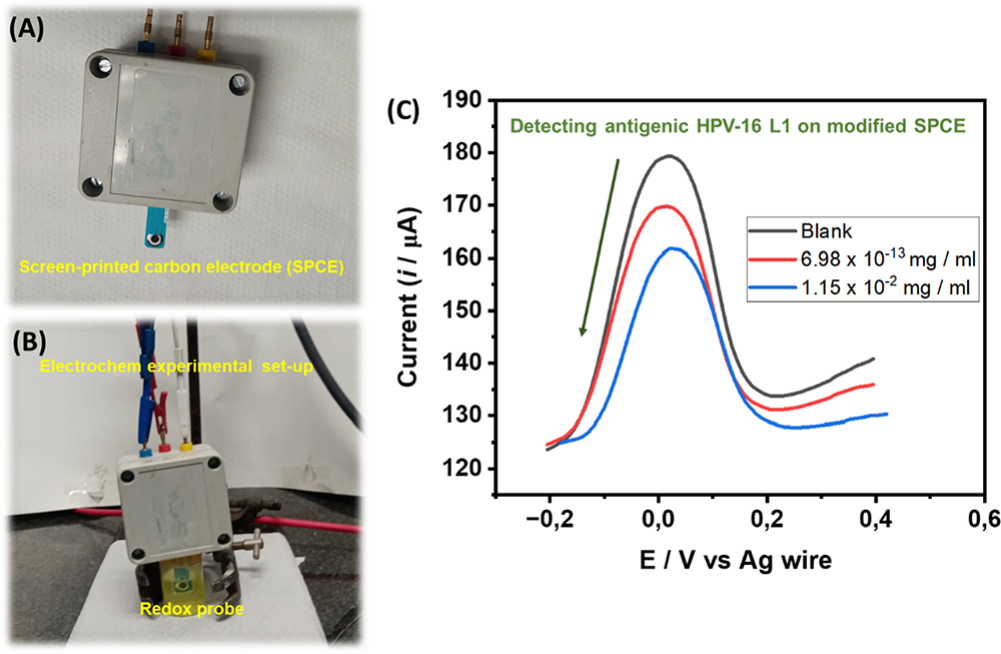
(A, B) Set-up of a screen-printed carbon electrode (SPCE) modified with HPV-16 L1 antibody immunosensor, (C) typical square wave voltammetric responses toward antigenic HPV-16 L1 protein. Please see the Experimental Section for details.

The possible practical application of the HPV immunosensor was tested using a screen-printed carbon electrode (SPCE) modified with the HPV-16 L1 antibody (Figure 6A,B). Although this was preliminary work, it is evident from Figure 6C that the immunosensor can detect very low (6.98 × 10^–13^ mg/mL) and high (1.15 × 10^–2^ mg/mL) concentrations of the antigenic HPV-16 L1. This result promises, upon thorough optimization, to move from lab-based techniques to PoC diagnosis of HPV infections.

## Conclusions

OLC and OLC-PAN were successfully utilized as electrode platforms for the detection of HPV-16 L1 antigen and antibody. Both platforms exhibited a wide linear concentration range (1.95 fg/mL to 6.25 ng/mL) and ultra-low detection of 1.83 fg/mL (32.7 aM) and 0.61 fg/mL (10.9 aM) for OLC-PAN and OLC-based immunosensors, respectively. The high specificity of detection was proven by experimenting with an anti-ovalbumin antibody (anti-OVA) and native ovalbumin protein (OVA). An immobilized antigenic HPV-16 L1 peptide showed insignificant interaction with anti-OVA in contrast with the excellent interaction with the anti-HPV-16 L1 antibody. The sensor’s reusability was also tested, and a couple of runs were done, which showed that even after using the sensor several times, the sensor was still active although there was a slight decrease in the current response. The immunosensor still showed some activity after the 7th day of storage upon surface regeneration. The application of the immunosensor as a potential PoC diagnostic device was investigated with the SPEC, which showed the ability to detect ultra-low (ca. 0.7 fg/mL ≈ 12.5 aM) and high (ca. 12 μg/mL ≈ 0.21 μM) concentrations. This work represents the lowest detection limit ever reported for HPV-16 L1 using electrochemical and non-electrochemical methods The results open the door of opportunity for studying other electrode platforms and realizing PoC diagnostic devices for screening and testing of HPV biomarkers for cervical cancer.

## Experimental Section

### Materials

*N*,*N* dimethyl formamide (DMF) was purchased from Merck. Ethanol and Nafion were purchased from Sigma Aldrich. Mono-potassium phosphate (KH_2_PO_4_), sodium chloride (NaCl), disodium phosphate (Na_2_HPO_4_), sodium hydroxide (NaOH), ethyl-3-(3-dimethylaminopropyl) carbodiimide (EDC), *N*-hydroxysuccinimide (NHS), ferro(IV) cyanide (K_4_[Fe(CN)6]·3H_2_O), and ferri(III) cyanide (K_3_[Fe(CN)6]·6H_2_O) and were purchased from Sigma Aldrich. Potassium chloride was purchased from Merck. The GCE was purchased from BASi. The carbon screen-printed electrodes were purchased from Metrohm. Recombinant HPV-16 L1 protein (HPV-16, 56 kDa), anti-HPV16 L1 antibody [Cam Vir 1] (anti-HPV-16, 56 kDa), anti-ovalbumin antibody (anti-OVA), and native ovalbumin protein (OVA) were purchased from Celtic molecular diagnostics (Pty) Ltd. Millipore water was obtained from Milli-Q Water Systems (Millipore Corp. Bedford, MA, USA). The saline buffer used was prepared at pH 7.4. OLC-PAN was synthesized according to the literature.^22^

### Instrumentation

The microstructures were analyzed by scanning electron microscopy (SEM, FEI Nova Nanolab 600). Elemental distribution was determined by energy-dispersive X-ray spectroscopy on an FEI Nova Nanolab 600. The crystal structures of the synthesized catalysts were investigated by powder X-ray diffractometer (D2 Phaser in Bragg–Brentano configuration equipped with a sealed Co *K*α (Å) radiation tube and a secondary beam of Fe *K*β filter, Bruker Lynxeye PSD detector, with primary and secondary Soller slits) in the 2θ range of 5–90°. Electrochemical experiments were performed with SP300 Potentiostat (BioLogic Science instrument running on EC-Lab software).

### Electrochemical Analysis

All electrochemical experiments were performed with bare or modified GCE (diameter = 3.0 mm, BASi) as the working electrode, platinum (Pt) rod as a counter electrode and the potential was measured against the Ag/AgCl electrode (saturated with 3 M KCl) used as the reference electrode. The GCE was first cleaned by polishing with alumina (Al_2_O_3_) slurry (50 nm powder), washed with ultrapure water, and sonicated in ethanol to remove slurry residues prior to use. Inks were prepared by mixing 1 mg of the electrocatalysts in 1 mL of DMF, and 20 μL of Nafion solution (5 wt %) and were ultrasonicated for 1 h to form a dispersed solution. The pre-cleaned GCE was then coated with 10 μL of the ink suspension and left to dry in an oven for 15 min at 30 °C. Every solution used in this work was obtained using ultrapure water (18.2 M Ω cm resistivity). Phosphate buffer solution containing a small amount of sodium azide (as a preservative) and ethylenediaminetetraacetic acid (PBS/AE, pH 7.4) was prepared as previously described^42,43^ and used to prepare the solutions of the HPV-16 L1 antigen and antibody. Redox probe solution of 0.1 mM K_4_Fe(CN)_6_/K_3_Fe(CN)_6_ (1:1 mixture) in PBS/AE (pH 7.4) was used for investigating the electrochemical performance of the fabricated immunosensor electrodes.

### Synthesis of OLC and OLC-PAN

#### Synthesis of OLC

OLC was synthesized from nanodiamond. Briefly, the nanodiamonds were placed in a sealed cylindrical graphite crucible and were thermally annealed in a water-cooled high-temperature vacuum furnace comprising tungsten heaters. The heating and the cooling rates were 15 °C/min, and the chamber pressure ranged between 10 and 100 mPa. To get the final OLC, further annealing at 1300 °C for 3 h under argon at 1 L/min was conducted.

#### Synthesis of OLC-PAN

In two separate conical flasks, 2 g of OLC and PAN were dispersed in 15 mL of DMF, respectively, after which the two solutions were mixed and stirred for 2 h at ambient temperature to form a uniform solution. The solution was further sonicated for 20 min to obtain a homogeneous mixture. The resulting polymer solution was fed into a syringe for electrospinning purposes. For electrospinning, the feed rate was 0.4 mL/h and 15 cm distance from the tip to the collector, while the potential difference between the collector and the grounded plate was 10 kV. The collected fibers were put in water overnight, to remove DMF and were then dried at 60 °C for 2 h in an oven.

### Fabrication of the Immunosensor

Figure 3 summarizes the fabrication of the sensor. GCE was modified using the drop casting method following a reported method.^23,33,34^ Briefly, 10 μL of electrocatalyst was drop-casted onto GCE and was dried in an oven at 45 °C for 30 min, and this was labeled as **GCE-OLC-PAN**. The **GCE-OLC-PAN** was functionalized with 3 M NaOH for 2 h, the electrode was washed with Millipore water and PBS, after which the functionalized electrode was dried with N_2_ gas, to afford a carboxylic acid functionalized surface (GCE-OLC-PAN-COOH). The COOH functional groups were activated using EDC and NHS pair (1:1% v/v) for 2 h with the above-stated conditions. After the activation of the COOH moiety, the HPV-16 L1 antibody containing NH_2_ groups was allowed to interact with the activated **GCE-OLC-PAN-COO**– overnight to afford a GCE-OLC-PAN-Ab. BSA was then used to block unreacted sites to afford the **GCE-OLC-PAN-Ab-BSA** sensor. The electrode was stored at 4 °C when not used. The screen-printed electrode was modified using the procedure described in Figure 3.

## Supporting Information Available

SAED, TEM images, TGA, Raman, coulombic efficiency and cycling stability, and the DFT determined structure (PDF)
