# Supplementary material for: Electrochemical Immunosensor for Ultra-Low Detection of Human Papillomavirus Biomarker for Cervical Cancer
Source: ACS Sens. 2023 Jun 29;8(7):2761–70. doi: 10.1021/acssensors.3c00677 (PMC10391710; doi:10.1021/acssensors.3c00677)
Supplement: Supplementary file 1 — se3c00677_si_001.pdf [file se3c00677_si_001.pdf]

SUPPORTING INFORMATION

Electrochemical Immunosensor for Ultra-low Detection of Human Papillomavirus Biomarker for Cervical Cancer

Siwaphiwe Peteni[1], Okoroike C. Ozoemena[1#], Tobile Khawula[1], Aderemi B. Haruna[1], Frankie J. Rawson[2], Leshweni J. Shai[3], Oluwafunmilayo Ola[4*], and Kenneth I. Ozoemena[1,*]

[1]*Molecular science Institute, School of Chemistry, University of the Witwatersrand, Johannesburg, South Africa*
[2]*School of Pharmacy, Biodiscovery Institue, University of Nottingham, Nottingham, UK*
[3]*Department of Biomedical Sciences, Tshwane University of Technology, Pretoria, South Africa*
[4]*Advanced Materials Group, Faculty of Engineering, The University of Nottingham, Nottingham, UK*

---

[*]Authors to whom correspondence should be addressed: O. Ola (email: Oluwafunmilola.Ola@nottingham.ac.uk; and K.I. Ozoemena (e-mail: Kenneth.ozoemena@wits.ac.za).

The author was a visiting student to the School of Chemistry, University of the Witwatersrand during this work. He has relocated to the Department of Chemistry, University of Guelph, Ontario, Canada.

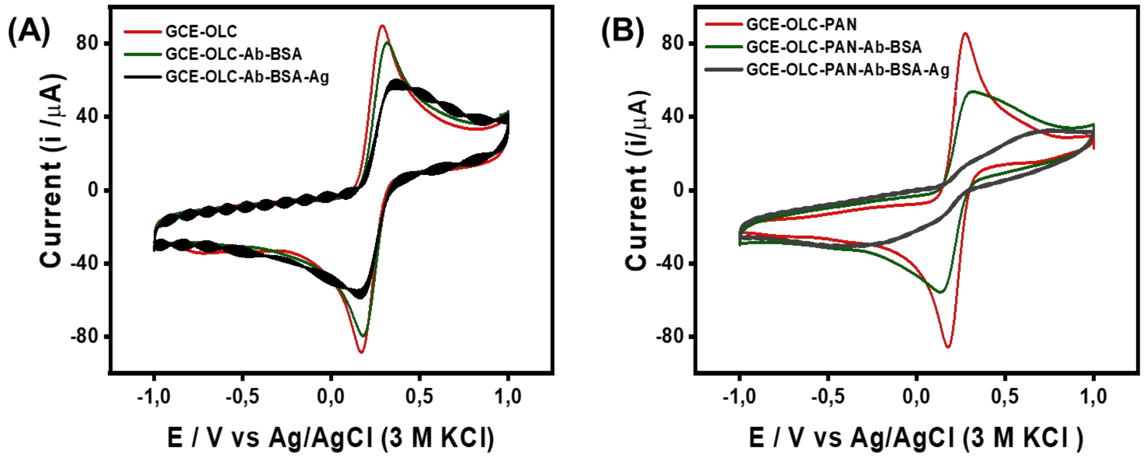

Fig. S1:Comparative cyclic voltammograms before and after antigen-antibody complexation: (A) OLC-based electrode and (B) OLC-PAN-based electrodes. All data were collected with HPV-16 L1 antigen (1.96 x 10^{-12} mg/mL) in the redox probe (0.1 mM $[Fe(CN)_6]^{4-}$/$[Fe(CN)_6]^{3-}$ in PBS/AE, pH 7.4) at 20 mVs^{-1}.
